# IFN-γ fails to overcome inhibition of selected macrophage activation events in response to pathogenic mycobacteria

**DOI:** 10.1371/journal.pone.0176400

**Published:** 2017-05-15

**Authors:** Shyamala Thirunavukkarasu, Karren M. Plain, Auriol C. Purdie, Richard J. Whittington, Kumudika de Silva

**Affiliations:** The University of Sydney School of Veterinary Science, Faculty of Science, The University of Sydney, Sydney, Australia; Cornell University, UNITED STATES

## Abstract

According to most models of mycobacterial infection, inhibition of the pro-inflammatory macrophage immune responses contributes to the persistence of bacteria. *Mycobacterium avium* subsp. *paratuberculosis* (MAP) is a highly successful pathogen in cattle and sheep and is also implicated as the causative agent of Crohn’s disease in humans. Pathogenic mycobacteria such as MAP have developed multiple strategies to evade host defence mechanisms including interfering with the macrophages’ capacity to respond to IFN-γ, a feature which might be lacking in non-pathogenic mycobacteria such as *M*. *smegmatis*. We hypothesized that pre-sensitisation of macrophages with the pro-inflammatory cytokine IFN-γ would help in overcoming the inhibitory effect of MAP or its antigens on macrophage inflammatory responses. Herein we have compared a series of macrophage activation parameters in response to MAP and *M*. *smegmatis* as well as mycobacterial antigens. While IFN-γ did overcome the inhibition in immune suppressive mechanisms in response to MAP antigen as well as *M*. *smegmatis*, we could not find a clear role for IFN-γ in overcoming the inhibition of macrophage inflammatory responses to the pathogenic mycobacterium, MAP. We demonstrate that suppression of macrophage defence mechanisms by pathogenic mycobacteria is unlikely to be overcome by prior sensitization with IFN-γ alone. This indicates that IFN-γ signaling pathway-independent mechanisms may exist for overcoming inhibition of macrophage effector functions in response to pathogenic mycobacteria. These findings have important implications in understanding the survival mechanisms of pathogenic mycobacteria directed towards finding better therapeutics and vaccination strategies.

## Introduction

Pathogenic mycobacteria are responsible for considerable mortality and morbidity in human and domestic livestock populations worldwide. A characteristic feature of this group of pathogens is their ability to cause chronic infection, often associated with similar immunopathological features, in a wide variety of host species [1, 2]. *Mycobacterium avium* subsp. *paratuberculosis* (MAP) is the causative agent of a chronic enteropathy in ruminants leading to death, termed paratuberculosis or Johne’s disease, and has also been implicated as a possible etiological agent of Crohn’s disease in humans [3–8]. Recently MAP was detected in the human population in a region of northern India with endemic Johne’s disease, at times associated with clinical enteritis [9, 10]. MAP has been suggested to also play a role in a range of other diseases such as thyroiditis, multiple sclerosis, type 1 diabetes and autism, due to the mechanism of antigenic mimicry, thus making this organism an intriguing potential zoonotic agent [11, 12]. Since MAP has been isolated from food sources for human consumption including dairy products, meat and drinking water [13–16] there is a need to fully understand the pathogenesis of this organism and devise strategies to eliminate it from the food chain.

Macrophages, apart from their innate immune functions like nitric oxide production, pathogen recognition and phagocytosis, apoptosis and secretion of cytokines, are also target cells that respond to cytokines such as interferon gamma (IFN-γ) resulting in the initiation of adaptive immune responses via major histocompatibility complex (MHC)-mediated processing and presentation of antigens [17]. The interplay between IFN-γ and macrophages in infection and inflammation involves a complex mechanism dependent on both host- and pathogen-associated factors and is fundamental to the host’s ability to control a pathogen [18].

Mycobacteria are highly successful pathogens of humans and animals and the obligate pathogenic strains have developed multiple strategies to evade host defence mechanisms and survive intracellularly in cells such as the macrophage. Therefore, the cell signaling pathways initiated by pathogenic mycobacteria such as MAP are likely to differ from non-pathogenic strains such as *M*. *smegmatis* or *M*. *phlei* [19–21]. One of the survival strategies of pathogenic mycobacteria includes interfering with the host macrophages’ capacity to respond to IFN-γ; both the whole bacterium and sub-cellular components of mycobacteria have the potential to inhibit macrophage immune responses to IFN-γ via toll-like receptor (TLR)2 dependent or independent mechanisms [22, 23].

Despite the success of these immune evasion strategies, *in vitro* studies have shown that if IFN-γ stimulation precedes infection, this can prime macrophages to initiate a pro-inflammatory phenotype and potentiate anti-bacterial mechanisms. Macrophage immune responses such as activation of inducible nitric oxide synthase (iNOS), tumour necrosis factor-alpha (TNF-α) production as well as nuclear factor kappa B (NFκB) activation in response to stimuli such as lipopolysaccharide (LPS), TLR agonists and bacterial DNA are enhanced upon IFN-γ priming [24–29]. Moreover, IFN-γ and TNF-α signaling pathways act synergistically, as many genes induced by IFN-γ are also induced by TNF-α such as *IRF-1* [30, 31]. Therefore, we hypothesize that these mechanisms of increased cellular activity due to IFN-γ pre-sensitization could help in overcoming the inhibition of inflammatory/protective responses that occur during infection with pathogenic mycobacteria such as MAP.

RAW 264.7 is a murine macrophage cell line which is extremely sensitive to stimulants [32]. It is an immortal cell line that grows continuously in culture due to alteration of genes which might also affect functional properties such as signalling cascades affected by microbial ligands [33, 34]. Despite this drawback, several studies have utilized the RAW 264.7 cell line for studying nitric oxide production in response to mycobacterial infections [35–37] and have reported differences in the immune responses and gene expression elicited between non-pathogenic and pathogenic mycobacteria or variant forms of mycobacteria such as cell-wall deficient forms in murine macrophages [19, 20, 38, 39]. A bovine SV40 transformed peritoneal macrophage cell line available for *in vitro* studies using mycobacteria, however detailed studies addressing the characteristics of this line are lacking [40, 41]. Although primary cells isolation from the blood of natural hosts for MAP like cattle and sheep is possible, the availability of reagents for functional studies in murine cells as well as the ease of culturing [42] makes cell lines such as RAW 264.7 an attractive alternative model for conducting studies. Therefore, we utilized the RAW 264.7 macrophage cell line as a model system to examine the effect of IFN-γ priming on the modulation of a comprehensive array of macrophage activation indices in response to a pathogenic mycobacterium (MAP), a non-pathogenic mycobacterium (*M*. *smegmatis*) as well as whole-cell derived and purified mycobacterial antigens. We demonstrate that the suppression of macrophage immune mechanisms by live pathogenic mycobacteria is unlikely to be overcome by prior sensitization with IFN-γ alone. Also, macrophages exhibit differential immune responses when exposed to whole mycobacteria in comparison to mycobacterial antigens, which should be borne in mind when trying to integrate antigenic mechanisms to explain mycobacterial pathogenesis.

## Materials and methods

### Macrophage cell culture and antigenic stimulation

RAW 264.7, a mouse macrophage cell line, was obtained from the European Collection of Cell Cultures and grown at 37°C in 5% CO_2_ in DMEM (Gibco) supplemented with 10% FCS (Invitrogen), penicillin at 100 U/mL and streptomycin at 100 μg/mL (Gibco) and β-mercaptoethanol (58 μM) (Sigma). Cells were plated 24 h prior to infection at 2 x 10^5^ cells/well in a 96-well tissue culture plate (Griener Bio-one). Some of the cultures were pre-treated with recombinant murine IFN-γ (Thermo Scientific) at 100 ng/mL or LPS (25 ng/mL) for one hour. Cultures were washed with fresh culture media and were treated with various antigens and mycobacterial strains (Table 1). MAP sheep strain (Telford 9.2), isolated and cultured in the laboratory as previously described [43] and *M*. *smegmatis* (a kind gift from Dr. Nicolas West, University of Sydney) were either used live or as heat inactivated (80°C for 1 hour) at a multiplicity of infection (MOI) of 100 to 1. In some experiments, *M*. *phlei* and *M*. *avium* were included as additional non-pathogenic strain controls at the same concentration mentioned above to assess the IFN-γ mediated macrophage response.

**Table 1:** Antigens and mycobacterial strains used for antigenic stimulation of macrophage cultures.

Antigens/bacteria used	Concentration	Source	Description
LPS	100 μg/ml	Sigma-Aldrich	Positive control (TLR4 agonist)
MAP 316v antigen	100 μg/ml	EMAI*	MAP cattle strain (316v) whole cell derived antigen
Tuberculin	10 μg/ml	Staten Serum Institute	*M*. *tuberculosis* antigen
PPDA	100 μg/ml	Prionics	*M*. *avium* PPD
PPDB	100 μg/ml	Prionics	*M*. *bovis* PPD
MAP S strain	100:1	In-house	Pathogenic strain (L&HK)
*M*. *smegmatis*	100:1	USyd	Non-pathogenic strain (L&HK)

LPS: lipopolysaccharide; Ag: Antigen; PPD: Purified protein derivative; L & HK: Live and heat-killed; USyd: University of Sydney.

* Kind gift from Elizabeth Macarthur Agricultural Institute (EMAI), Department of Primary Industries, New South Wales, Australia.

Supernatants from triplicate cultures from the different treatment groups were harvested at 48 h post-incubation and stored at -80°C until further analysis. Cells were harvested at 24 h post-incubation for flow cytometry, RNA extraction and fluorescence microscopic studies.

### Detection of macrophage functions

#### Nitric oxide production

Estimation of nitric oxide production was carried out on the stored cell culture supernatants by the Griess assay kit (Sigma) according to the manufacturer’s instructions.

#### Cytokine production

Cytokine levels in culture supernatants were assessed using the FlowCytomix multiplex cytokine assay kit^®^ (eBioscience), performed according to the manufacturer’s protocol. Flow cytometry data were acquired on a FACSCalibur (Becton-Dickinson) and analyzed using the FlowCytomix Pro^®^ software (eBioscience).

#### Activation and translocation of NFκB

Murine (RAW264.7) macrophages were cultured in 4-well chamber slides (Chamber slide^™^ system, Nunc^®^, Denmark) at 4 x 10^5^ cells/chamber in triplicate. Pre-treatment and incubation with antigens/mycobacteria (Table 1) was carried out for 30 minutes (based on a series of optimization experiments to determine the optimum minimum time of incubation to generate appreciable NFκB cytoplasmic to nuclear translocation). Immunofluorescence staining of the cells was carried out using the Cellomics^®^ NFκB activation kit (Thermo Fisher Scientific, Waltham, MA) following the manufacturer’s instructions. Briefly, cells were fixed with 4% paraformaldehyde, permeablized and incubated with rabbit anti-NFκB polyclonal primary antibody for 1 h. The cells were then incubated with DyLight^™^488 goat anti-rabbit secondary antibody and Hoechst dye. Visualization was by an Olympus^®^ BX61 fluorescence microscope. Image analysis was by ImageProPlus v 5.0 software (Media Cybernetics). The nuclear to cytoplasmic fluorescence intensity was calculated by dividing nuclear fluorescence by cytoplasmic fluorescence. The entire process was repeated for 6 images (two sets for each replicate chamber). The average nucleus: cytoplasm (N: C) NFκB fluorescence was then calculated for each treatment group and data plotted as a histogram.

#### Assessment of RAW264.7 macrophage surface TLR2 and MHCII expression

RAW 264.7 cells were harvested in ice cold buffer (PBS with 2% newborn calf serum band 0.05% sodium azide) and labelled with rat monoclonal anti-mouse MHC class II non-polymorphic region (NIMR-4) FITC (Abcam), FITC-conjugated anti-mouse TLR2 (eBioscience) or respective isotype controls and fixed in 1% paraformaldehyde, as previously described [44]. Data were acquired on a FACSCalibur flow cytometer (BD Biosciences). Expression of MHC-II on the positively stained population (cells staining for the respective antibodies) was analysed using CellQuest Pro software (BD Biosciences). Data are presented as the median fluorescence intensity (MFI) for the specific antibody minus the MFI for the isotype control antibody.

### Gene expression

#### RNA extraction and genomic DNA removal

RNA extraction and cDNA synthesis were performed as previously described [45]. Briefly, RNA was extracted using the Mini RNeasy kit (Qiagen) according to the manufacturer’s protocol. Contaminating genomic DNA was removed by DNase digestion using 10 U of RQ1 DNase (Promega) for 2 h at 37°C. Precipitation of RNA was accomplished by addition of 0.1 volume of 3 M sodium acetate (pH 5.3), 3 volumes of ethanol (Sigma) and 0.5 μg linear acrylamide (Ambion). The mixture was incubated for 60 minutes on dry ice, centrifuged 16,000 x *g* for 45 min at 4°C. This was followed by purification of the RNA samples by incubating with ethanol (Sigma) and 0.3 M linear acrylamide. The purified RNA was re-dissolved in 50 μL of nuclease-free water. Quantity and integrity of RNA was verified by spectrophotometry (Nanodrop). The quality was assessed by spectrophotometric quantification (A_260nm_/A_280nm_ and A_260nm_/A_230nm_ ratios).

#### Panomics assay

The QuantiGene 2.0 Plex Assay^®^ (Panomics Solutions, Affymetrix, Santa Clara, CA), a branched DNA (bDNA) hybridisation based method of target-specific RNA quantitation by signal amplification using labelled DNA probes, was used to target RNA for genes of interest (Table 1) following the manufacturer’s protocol (http://cdn.panomics.com/downloads/UM13074_QG2Manual_RevC_100111.pdf). Briefly, target hybridization was carried out by incubating RNA samples with a mixture of Luminex beads coated with capture probes, capture extenders, label extenders and blocking probes. This was followed by the signal amplification step where the hybridized RNA was incubated with pre-amplifier, amplifier and label probe. Detection was by addition of streptavidin phycoerythrin and signals were read using the Luminex^®^ flow cytometer (Life Technologies). Signal is reported as MFI and is proportional to the number of target RNA molecules in the sample. Gene expression was calculated as a fold change in the expression of the gene of interest between the negative untreated and treated samples as detailed by the manufacturer’s protocol. To do this, firstly the average background signal was deducted from MFI for the different genes. If this value was ≤ 0, then the value was set to the minimum fluorescence detectable (1) so that calculations of fold change could be performed. The MFI result for individual wells was then normalized by dividing by the respective reference gene MFI. The fold change was then calculated for each test gene by dividing the average values for the treated samples in each treatment group by the corresponding average values obtained for those genes in the media control without IFN-γ pre-treatment.

### Statistical analysis

Data were analyzed for differences between pre-treatment groups and between the various stimulants used. This was carried out by Restricted Maximum Likelihood (REML) analysis in a linear mixed model (GenStat, 13th edition, VSN International Ltd, UK) to determine statistical significance at the 5% level. Predicted means were generated by the model and were considered significantly different to each other if they varied by an amount greater than the Least Square of Differences [46]. In most data sets, a log_e_ transformation was first undertaken to normalize the data. Data were tabulated according to the different pre-treatment groups as well as the different stimulants used in the assays. Both were fixed effects for the model. Experiments repeated or individual replicate wells were categorized as a random effect, to account for repeated measures.

Data for the multiplex cytokine array experiment were collected in duplicate and results were averaged and reported as the mean plus standard error of the mean. Data were analyzed by use of a general analysis of variance (ANOVA) followed by the Bonferroni multiple comparison test. The means of interest were compared by the student’s t-test. *P* ≤ 0.05 was considered to be significant (Genstat 13.0^®^ software).

## Results

### Modulation of cellular responses by mycobacterial antigens in response to prior sensitization of cells with IFN-γ

The optimum dose of PPDA, PPDB and MAP cattle strain 316v whole cell-derived antigen (316v Ag) was selected by titration (0–128 μg/mL), following pre-treatment of RAW 264.7 murine monocytic cells for one hour with IFN-γ at a final concentration of 100 ng/mL. An increase in nitric oxide production in response to the mycobacterial antigens tested was observed from 32–128 μg/mL (S1A–S1D Fig). However, the overall nitric oxide response to MAP 316v Ag was relatively low compared to the other mycobacteria-derived antigens tested. The positive control, LPS, consistently induced nitric oxide (S1 Fig), while background nitric oxide production was seen in the media control.

**Fig 1:**
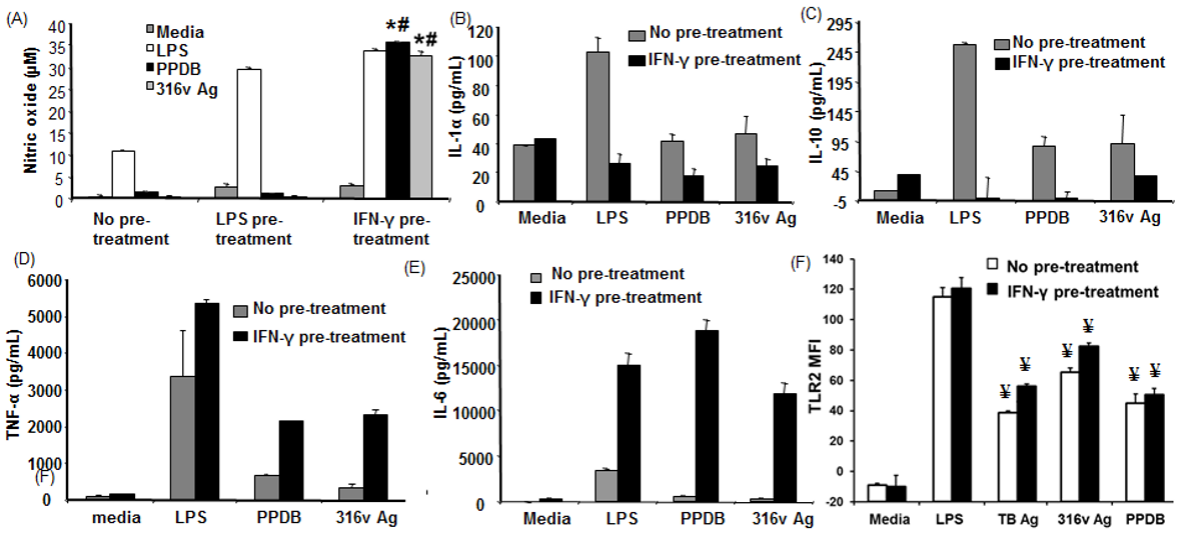
Effects of IFN-γ pre-sensitization on the macrophage effector responses to mycobacterial antigens. RAW 264.7 cells received either no pre-treatment or were pre-treated with LPS (1 ng/mL) or IFN-γ (100 ng/mL) for one hour and then incubated with LPS (25 ng/mL), 316v or PPDB (100 μg/mL) for a period of 48 h. Supernatants were harvested and assessed for (A) nitric oxide or the cytokines (B) IL-1α, (C) IL-10, (D) TNF-α, (E) IL-6 and (F) TLR2 expression presented as change in the median fluorescence index (MFI). Data are the mean plus standard error of the mean of three replicate cultures from one of two independent experiments for (A), triplicate cultures from one experiment for (B-F). * denotes a significant difference (*P* ≤ 0.05) between the no pre-treatment and IFN-γ pre-treatment groups, ^#^ denotes a significant difference (*P* ≤ 0.05) between the LPS pre-treatment and IFN-γ pre-treatment groups. ^¥^ denotes significant difference in TLR2 expression (*P* ≤ 0.05) in response to antigenic stimulation compared to the respective media control.

Pre-treatment of RAW 264.7 cells with LPS or IFN-γ was compared, followed by stimulation with the mycobacterial antigens, PPDB and 316v Ag. Pre-treatment with IFN-γ led to significantly enhanced (*P* ≤ 0.05) nitric oxide production in response to mycobacterial antigen stimulation, however there was no induction of nitric oxide production by either of the antigens tested following LPS pre-treatment (Fig 1A). All the mycobacterial antigens tested induced some production of both pro- and anti-inflammatory cytokines, particularly TNF-α and IL-10. Following pre-treatment with IFN-γ, the production of pro-inflammatory cytokines (TNF-α and IL-6) was markedly enhanced while there was an apparent decrease in IL-10 levels (Fig 1C–1E). No changes were observed in IL-1α levels between the MAP 316v Ag and PPDB treatment groups in the absence of prior stimulation. However, IFN-γ pre-treatment resulted in a decrease in IL-1α levels in response to PPDB and 316v Ag when compared with the no pre-treatment group (Fig 1B); but these changes did not reach statistical significance.

**Fig 2:**
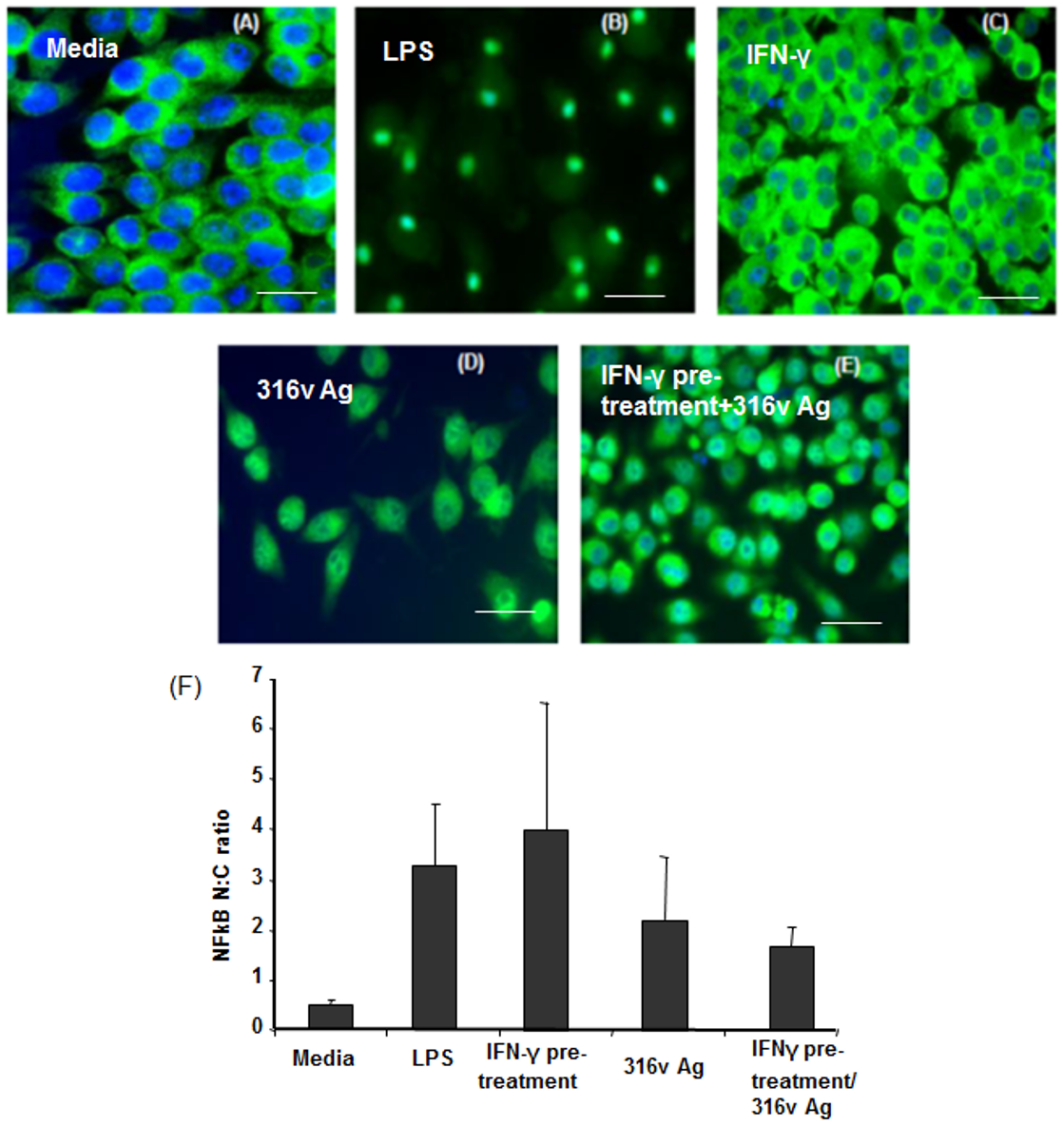
Effect of IFN-γ pre-sensitization on macrophage NFκB activation and translocation in response to MAP 316v antigen. RAW 264.7 cells were incubated for one hour with media (A) LPS (100 ng/mL) (B), IFN-γ priming alone (C), 316v Ag (100 μg/mL) without any pre-treatment (D) or 316v Ag (100 μg/mL) after pre-treatment with IFN-γ (100 ng/mL) for 30 minutes (E). The cells were stained for NFκB by immunofluorescence and images were captured using a fluorescence microscope. Image analysis and quantification was performed using using Image ProPlus^®^ software (F). Data is representative of triplicate treatments of one out of two separate experiments.

TLR2 surface expression was enhanced in response to exposure to mycobacterial antigen stimulation, when compared to the unstimulated media control (Fig 1F), however there was no significant difference between the different pre-treatment groups. Furthermore, although MAP 316v Ag stimulated the cells to activate NFκB, the degree of activation was lower in comparison to the positive controls LPS and IFN-γ. Pre-treatment with IFN-γ followed by 316v Ag stimulation did not further increase NFκB activation compared to the levels seen with 316v Ag alone (Fig 2). NFκB activation and translocation in response to live and heat killed MAP and *M*. *smegmatis* was also assessed with and without IFN-γ pre-treatment, but there were no significant differences between the treatments (S2 Fig).

### Modulation of cellular responses by prior exposure to mycobacterial antigens

**Fig 3:**
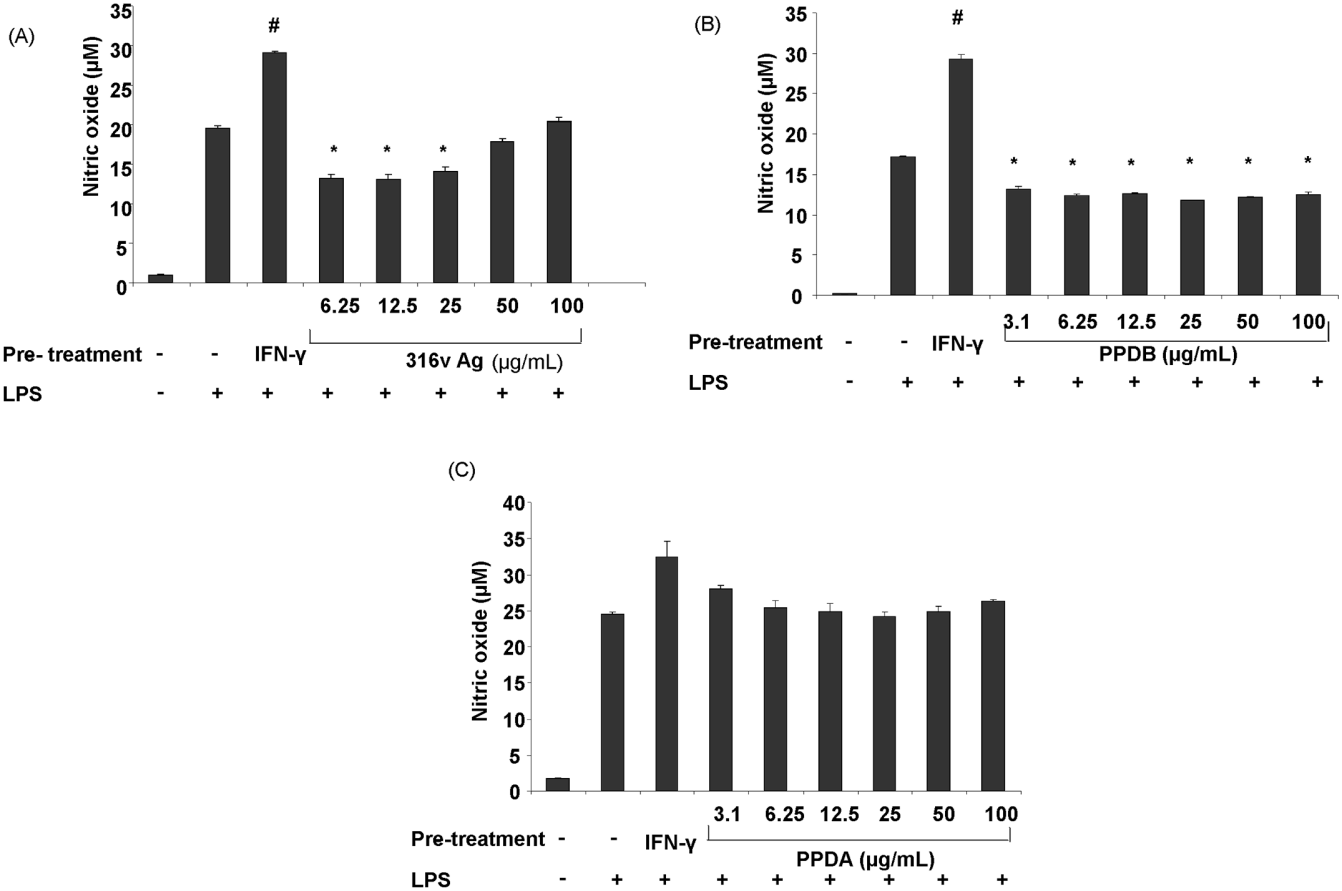
Suppression of macrophage nitric oxide production by pre-treatment with mycobacterial antigens. RAW 264.7 cells pre-treated with 316v (A), PPDB (B), PPDA (C) (6.25 to 100 μg/mL) or IFN-γ (100 ng/mL) concentration for one hour followed by incubation with LPS (25 ng/mL). Nitric oxide was determined in supernatants harvested at 48 h post-incubation. Data are the mean plus standard error of the means of five replicates from two independent experiments. * denotes a significant difference (*P* ≤ 0.05) between the LPS induced nitric oxide production with and without antigen pre-treatment. # denotes a significant difference (*P* ≤ 0.05) in LPS induced nitric oxide production after IFN-γ pre-treatment compared to the group without IFN-γ pre-treatment.

To compare the effect of the type of initial stimulus that macrophages receive on their downstream responses, RAW 264.7 cells were pre-treated with mycobacterial antigens (316v Ag, PPDB or PPDA) and their subsequent LPS-induced nitric oxide production response was assessed. Compared to no prior treatment, IFN-γ pre-treatment (positive control) significantly increased (*P* ≤ 0.05) the LPS-induced nitric oxide response (Fig 3). In contrast, pre-treatment with 316v Ag or PPDB significantly decreased nitric oxide levels produced in response to LPS: with the former, this was only at lower concentrations (*P* ≤ 0.05) (Fig 3A and 3B). Pre-treatment with purified protein derivative from the non-pathogenic mycobacterium, *M*. *avium* (PPDA), however, did not have an appreciable effect on the LPS responsiveness (Fig 3C).

### Differential responses to pathogenic versus non-pathogenic mycobacteria following priming of cells with IFN-γ

**Fig 4:**
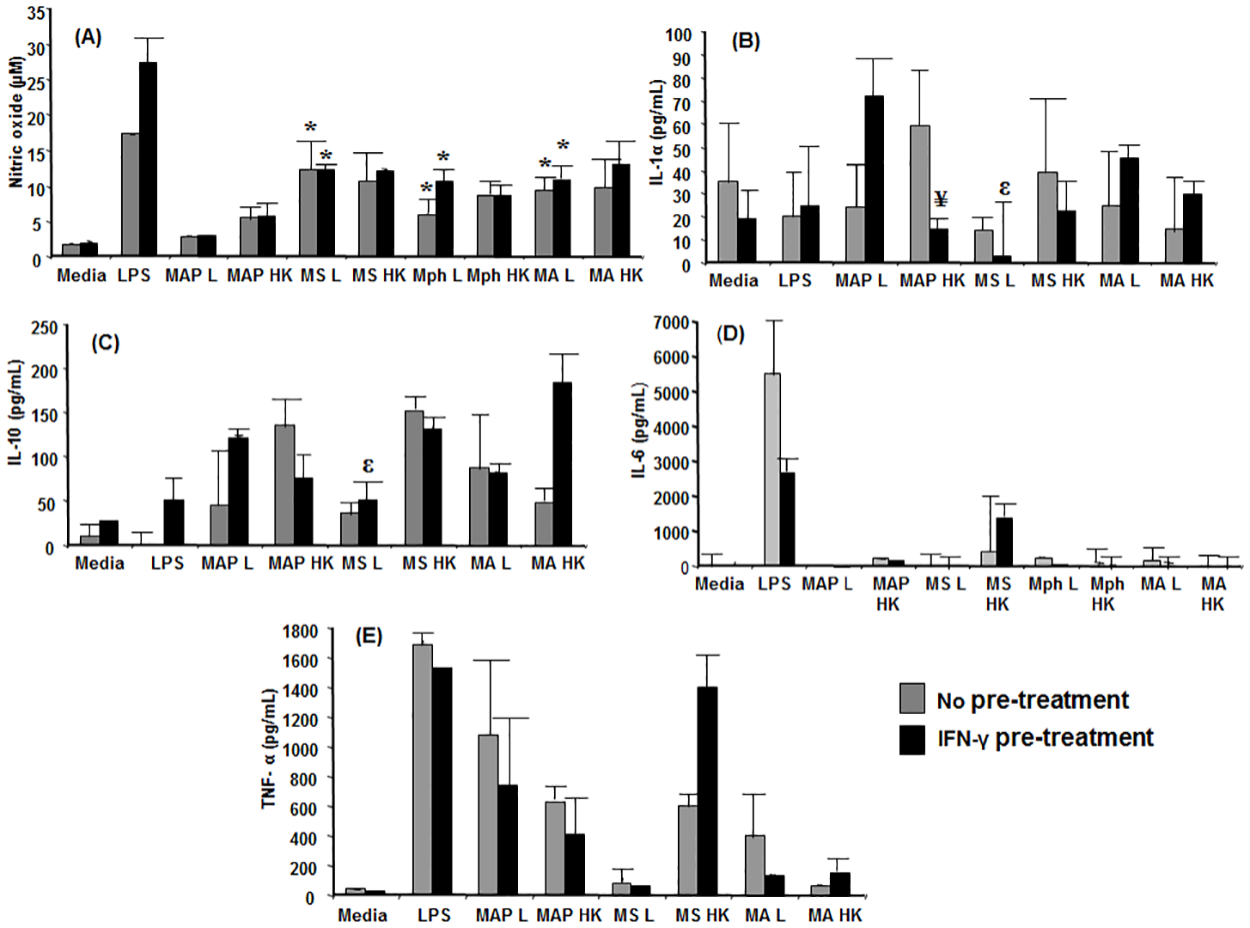
Effect of IFN-γ pre-sensitization on macrophage effector responses to MAP and non-pathogenic mycobacteria. RAW 264.7 cells were incubated with MAP or non-pathogenic mycobacterial strains, live (L) or heat-killed (HK), at an MOI of 100 to 1 for a period of 48 h with or without pre-treatment with IFN-γ. Supernatants were harvested and assessed for (A) nitric oxide, (B) IL-1α, (C) IL-10, (D) IL-6 and (E) TNF-α. Data are the mean plus standard error of three replicate cultures from one of two independent experiments for (A) and triplicate cultures from one experiment for (B-E). MS = *M*. *smegmatis*, Mph = *M*. *phlei*, Maa = *M*. *avium*. * denotes a significant difference (*P* ≤ 0.05) compared to MAP (L) in the respective no pre-treatment IFN- γ pre-treatment groups. ^ε^ is significant difference (*P* ≤ 0.05) between MAP (L) and MS (L) for the IFN-γ pre-treatment group. ^¥^ is significant difference between (*P* ≤ 0.05) MAP (L) and MAP (HK) for the IFN-γ pre-treatment group.

Nitric oxide production in response to all of the non-pathogenic live mycobacteria (*M*. *smegmatis*, *M*. *phlei* and *M*. *avium*), was significantly enhanced (*P*≤0.05) when compared to the nitric oxide response induced by pathogenic MAP (Fig 4A). IFNγ pre-treatment of macrophages did not enhance the nitric oxide response; in addition, there were no significant differences in nitric oxide production between live and heat-killed mycobacteria (Fig 4A).

In cells that were pre-treated with IFN-γ, IL-1α production in response to live MAP was significantly greater than in response to heat-killed MAP (*P* ≤ 0.05) (Fig 4B). There was also a significant increase in IL-1α production in response to live MAP compared to live *M*. *smegmatis* in the IFN-γ pre-treatment group (*P* ≤ 0.05) (Fig 4B). Similarly, there was a significant increase in IL-10 production between live MAP and live *M*. *smegmatis* in response to IFN-γ pre-treatment (*P* ≤ 0.05) (Fig 4C). There were no significant differences in the IL-10 production in response to heat-killed organisms in comparison to live organisms for the different mycobacterial species tested (Fig 4C).

No significant difference in IL-6 or TNF-α production was observed for the different treatment groups (Fig 4D and 4E).

### Gene expression profile following exposure to mycobacteria and their antigens in response to priming of cells with IFN-γ

The effect of pre-stimulation with IFN- γ on differential expression of genes when macrophages are stimulated with live mycobacteria or mycobacterial antigens was assessed. RAW264.7 macrophages were left untreated (media alone) or pre-treated with IFN-γ (100 ng/mL) for one hour and then incubated with live or heat-killed mycobacteria or mycobacterial antigens for 24 h. Expression of 10 genes of interest and three reference genes was determined using the Quantigene 2.0 Plex assay. The results obtained for the genes related to nitric oxide production (*iNOS*), IFN-γ signaling (*IRF-1* and *IFNGRA-1*), apoptosis (*FAS*) and production of other pro-inflammatory mediators like cytokines and chemokines are presented in the form of a heat map (S3 Fig) and as a Table for ease of interpretation (Table 2).

**Table 2:** Effect of IFN-γ pre-sensitization of macrophage gene expression in response to MAP, non-pathogenic mycobacteria and mycobacterial antigens.

Treatment		*iNOS*	*IFNGRA*	*IRF-1*	*TNF*	*IL-1α*	*IL-10*	*IL-6*	*IL-12b*	*FAS*
**No pre-treatment**	Media	-	-	-	-	-	-	-	-	-
	LPS	145.42	-1.51	1.52	3.66	186.65	-	1168.36	-	12.86
	316v	21.56	-	-1.52	1.66	4.85	-	8.99	-	4.15
	PPDB	70.08	-1.53	-	2.50	10.2	1.53	90.77	-	6.61
	Mptb Live	-	-	-	-	4.84	-	-	-	-
	Mptb HK	-	-	-	-	-	-	1.62	-	-
	MS live	4.02	-	-	-	4.48	-	-	-	3.95
	MS HK	4.42	-	-	-	3.17	-	1.69	-	2.55
**IFN-γ pre-treatment**	Media	2.08	-	2.81	-	-	-	-	10.74	1.94
	LPS	113.32	-	2.63	4.12	146.76	-	1195.09	58.23	13.19
	316v	42.24	-	-	1.81	6.35	-	21.95	-2.50	6.36
	PPDB	168.78	-1.53	-	2.24	19.73	1.86	258.71	18.72	6.41
	Mptb Live	-	-	-	-	3.51	-	-	-	1.51
	Mptb HK	-	-	-	-	3.88	-	-	3.44	-
	MS live	4.61	-	-	-	3.80	-	-	-2.12	4.26
	MS HK	4.74	-	-	-	3.54	-	-	-7.89	2.54

Results are expressed as fold change compared to media with no pre-stimulation. Negative values indicate downregulation. Changes <1.5 fold were considered to be not regulated. These are shown as “-“and were comparable to the media control.

*iNOS* expression generally increased after IFN-γ pre-treatment in response to mycobacteria and antigens, with the exception of live and heat-killed MAP where there was no increase in expression compared to the unstimulated media controls. Live and heat-killed non-pathogenic *M*. *smegmatis* caused the induction of *iNOS* expression with and without IFN-γ pre-treatment while pathogenic MAP did not. Although the expression of *iNOS* increased after IFN-γ pre-treatment in response to incubation with 316v antigen, the fold change was lower in comparison to the other mycobacterial antigen tested, PPDB (S3 Fig and Table 2).

Similar to the induction seen for *iNOS* gene expression, LPS induced high levels of cytokine and chemokine receptor gene expression, including *IL-1α*, *IL-1β* and *IL-6* but *TNFα* was induced to a lower extent (>1000 fold on some cases). In contrast, live or heat-killed MAP did not induce these genes (with or without IFN-γ pre-stimulation), with the exception of a small increase in *IL-1α*. Responses to 316v Ag were reduced for the cytokines tested in comparison to PPDB antigen, similar to the findings for the expression of *iNOS* (S3 Fig and Table 2).

*IRF-1*gene expression increased after pre-treatment with IFN-γ for the media control and the positive control, LPS. However, for the mycobacterial antigens tested as well as live and heat killed MAP and *M*. *smegmatis* there was almost no differential regulation of *IRF-1*. *IFNGRA-1* expression did not show any induction in response to incubation with the different antigens or mycobacteria with or without IFN- γ pre-treatment (S3 Fig and Table 2).

For the expression of the *FAS* gene, it was noticed that LPS stimulation caused moderate levels of induction. The mycobacterial antigens tested and both live and heat-killed *M*. *smegmatis* also caused induction of *FAS* gene though the levels were comparatively lower. In contrast, live MAP did not cause induction of this gene in the cultures without any pre-treatment. After IFN-γ pre-treatment this gene was induced in response to incubation with live MAP, although the fold change was only just greater than the 1.5 fold threshold applied (S3 Fig and Table 2).

### Role of IFN-γ in modulating the expression of MHC-II in response to mycobacteria and 316v antigen

**Fig 5:**
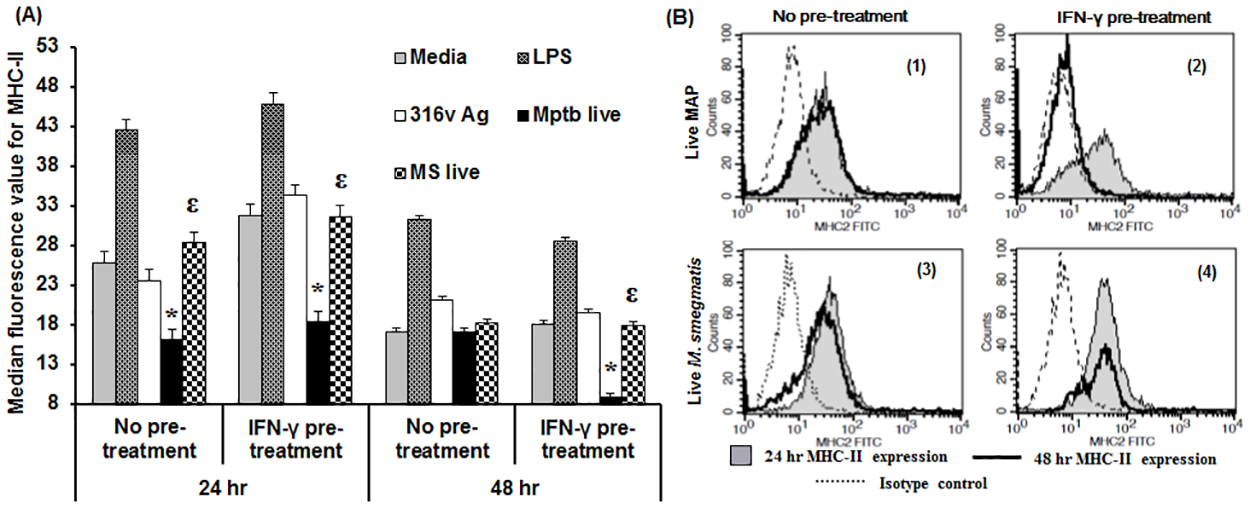
Effect of IFN-γ pre-sensitization on macrophage MHC-II expression in response to pathogenic mycobacteria. RAW 264.7 macrophages were incubated with 316v Ag (100 μg/mL), live MAP or *M*. *smegmatis* at an MOI of 100:1 with or without or IFN-γ (100 ng/mL) pre-treatment for one hour. Surface expression of MHC-II was detected by flow cytometry. Median MHC-II fluorescence minus the median for the isotype control was used to represent MHC-II expression (A). Data shown are the mean plus standard error of the mean for receptor expression for three replicate cultures. * denotes significant difference (*P* ≤ 0.05) in MHC-II expression between cultures incubated with live MAP compared to the media control and ^ε^ denotes significant difference (*P* ≤ 0.05) in MHC-II expression between cultures incubated with live MAP compared to cultures incubated with live *M*. *smegmatis*. Representative histograms are also presented (B) for cells with live MAP without (B1) and with IFN-γ pre-treatment (B2) and live *M*. *smegmatis* without (B3) and with IFN-γ pre-treatment (B4). At 24 h (shaded histogram), 48 h (thick black outline) and the isotype control (dotted line).

Another macrophage activation parameter, MHC-II expression, was assessed at 24 and 48 h after incubating cells with LPS, live MAP or *M*. *smegmatis*. IFN-γ pre-treatment increased the expression of MHC-II within 24h; this was particularly evident in the mycobacterial antigen stimulated cultures (S4 Fig). The positive control, LPS, induced the highest MHC-II expression (S4 Fig and Fig 5A). MHC-II expression was suppressed in response to live MAP at 24 h post-incubation and at 48 h post-incubation, MHC-II expression in response to live MAP was significantly reduced in the group with IFN-γ pre-treatment. There was a significant decrease in MHC-II expression in response to live MAP in comparison with live *M*. *smegmatis* with and without IFN-γ pre-treatment at 24 h and in the IFN-γ pre-treatment group at 48 h (Fig 5A and 5B). Live *M*. *smegmatis* did not induce MHC-II expression at either time point (Fig 5A). Overall there was a decrease in MHC-II expression over time.

## Discussion

It has been shown that pre-treatment of macrophages with IFN-γ increases macrophage inflammatory responses which could confer protection against infections [47]. Therefore, in this study we intended to answer the question as to whether prior sensitization of macrophages with IFN-γ could alter macrophage activation parameters in response to MAP or its antigens. We further compared these responses to non-pathogenic mycobacteria. We have found that while IFN-γ pre-treatment could selectively overcome the inhibitory effect to mycobacterial antigens, including MAP 316v Ag, non-pathogenic *M*. *smegmatis* as well as heat-killed MAP, on some parameters, it did not have any effect on the macrophage effector responses to live MAP.

We demonstrate that the inhibitory action of MAP 316v Ag and *M*. *bovis* PPDB on nitric oxide production could be overcome by pre-treatment with IFN-γ but not LPS. The effect of IFN-γ pre-treatment was akin to a ‘switch’ that enabled the cells to go from no nitric oxide response to full responsiveness to these antigens. This shows selective responsiveness of macrophages to mycobacterial antigens if they are exposed to IFN-γ prior to encounter with these antigens. This altered response did not correlate with any change in the expression of TLR2, a two pattern recognition receptor known to be involved in recognition of mycobacterial antigens.

This response pattern was reversed if the first encounter of the macrophage is with antigens from pathogenic mycobacteria. A recent study has reported that PPE2 protein of Mtb was capable of translocating to the nucleus of host cells and thereby inhibiting NO production [48]. We observed a significant reduction in the responsiveness to LPS-induced nitric oxide production upon prior sensitization with 316v Ag and PPDB, but not PPDA in our study. This could be attributed to immune tolerance [49]. These findings are similar to that observed by Trajkovic et al. [50] using a secreted protein of *M*. *tuberculosis* (Mtb), wherein an Mtb specific antigen 10 (MTSA-10) was found to bind to the surface of J774 macrophage-like cells and induce nitric oxide in synergy with IFN-γ. However pre-treatment of cells with MTSA-10 or a whole cell lysate of Mtb caused a marked reduction in nitric oxide production upon further incubation with LPS. This reduction in nitric oxide production after pre-treatment with MTSA-10 was attributed to desensitisation of macrophages mediated by MAPKp38 activity. A similar mechanism involving MAPKp38 may be occurring upon pre-treatment with PPDB and MAP 316v whole cell-derived antigen, as this signalling pathway has been shown to be activated upon exposure to certain MAP lipoproteins [51]. It could be speculated that a similar effect may occur *in vivo*, which would possibly reduce the ability of macrophages pre-exposed to antigens derived from pathogenic mycobacteria like MAP and *M*. *bovis* to carry out nitric oxide-mediated killing of intracellular bacteria upon subsequent infection. However, lack of a similar response to *M*. *avium* PPDA indicates that tolerance could be a phenomenon related to the relative pathogenic potential of an organism. Assessing a time line of the capability of macrophages exposed to different mycobacterial antigens to inherently produce NO and the ability of IFN-γ to possibly alter this might provide further insight into the pathogenic potential of mycobacterial antigens.

In contrast to the mycobacterial antigens, IFN-γ pre-treatment did not increase the nitric oxide response to live or heat-killed mycobacteria. Nitric oxide levels produced in response to the non-pathogenic mycobacteria tested were significantly higher than upon incubation with live MAP. We also observed that heat-killed MAP was more potent in inducing nitric oxide production in comparison to live MAP. In certain bacterial diseases like *Helicobacter pylori* infection, live bacteria were shown to inhibit nitric oxide production, however heat-killed bacteria were able to stimulate nitric oxide production [52]. The reason attributed to this was that live bacteria suppress iNOS expression [52]. It has been established that in macrophages activated with LPS and IFN-γ, an actin dependent recruitment of iNOS occurs [53]. However, this is inhibited in macrophages infected with live mycobacteria. Downregulation of expression of EBP50, a scaffolding protein involved in binding of iNOS and linking it to the actin skeleton, is implicated as the mechanism for this and this phenomenon depends on the viability of the organisms [53]. This increase in nitric oxide response after prior treatment with IFN-γ in response to mycobacterial antigens could be mediated by iNOS as found in this study. Interestingly, this increase in *iNOS* expression in response to mycobacterial antigens after pre-treatment with IFN-γ was not correlated with increased expression of *IFNGRA-1* or *IRF-1* in this study. Examination of varying time-points of post-stimulation or other factors such as MAPKp38 activity should be assessed by future studies.

It is worthwhile to note that an increase in *iNOS* gene expression was noticed in cells incubated with only live *M*. *smegmatis* and not live MAP in this study. Moreover, we have previously published data showing significantly increased TLR2 expression in response to the pathogenic mycobacteria MAP in comparison to the non-pathogenic *M*. *smegmatis* even after IFN-γ pre-treatment [54]. This along with the data obtained in our current study leads us to speculate that decreased nitric oxide response in response to pathogenic mycobacteria could be mediated through TLR2 and is likely independent of the presence or absence of IFN-γ in the environment. Hence, it could be argued that the decreased capacity of MAP-infected macrophages to generate appreciable amounts of nitric oxide could be due to the inherent capacity of the live bacteria to inhibit the nitric oxide response in macrophages which cannot be overcome even with prior sensitization with IFN-γ.

We also found that MAP as well as its antigen 316v induces activation and translocation of NFκB and that IFN-γ pre-treatment does not cause a significant difference in this parameter. The results of this study agree with that of Weiss et al. [55], who reported that MAP induces activation and translocation of NFκB in bovine monocytes resulting in enhanced TNF-α and IL-10 secretion. It has been shown that an increase in NFκB has been associated with a decrease in apoptosis [56, 57]. This was validated in our study, in which the *FAS* gene was poorly induced in response to MAP and 316v Ag even after IFN-γ pre-treatment, in comparison to the induction seen by non-pathogenic *M*. *smegmatis*. This gene codes for the FAS death receptor (CD95) located on the surface of cells and is involved in promoting apoptosis. Pathogenic mycobacteria like *M*. *leprae* and Mtb upregulate host macrophage production of anti-apoptotic genes and prevent macrophage apoptosis *in vitro* depending on the MOI; apoptosis and cell death are enhanced at very high bacterial loads [58–62]. The interaction between FAS-ligand (FasL) and the FAS-receptor is critical for apoptosis to occur as FasL associates and trimerizes the FAS receptor on cell surfaces resulting in the initiation of extrinsic apoptosis pathways [63, 64]. Mycobacteria have been shown to increase expression of FAS-ligand and decrease expression of FAS receptor on infected macrophages and thus overcome apoptosis which is believed to be one of the mechanisms for its survival [58, 59, 65]. Moreover, autophagy which is another innate immune mechanism capable of eliminating mycobacteria is under the control of pattern recognition receptors such as TLR and is activated by Th1 cytokines including IFN-γ [66]. Some pathogenic mycobacteria such as the Beijing strain of Mtb are capable of suppressing JNK and p38 kinases and NFκB by exploiting host ubiquitination mechanisms [67, 68]. Although we did observe an increase in TLR2 expression in response to pathogenic mycobacterial antigens it was independent of IFN-γ pre-sensitization and therefore it is likely that MAP might alter autophagy also but this requires further targeted studies.

IFN-γ pre-treatment also failed to overcome the inhibition of another critical component of macrophage activation; namely, MHC-II expression, which is believed to be downregulated by pathogenic mycobacteria [69, 70]. Decreased MHC-II expression even after IFN-γ pre-treatment in response to incubation with pathogenic mycobacteria has also been reported previously [71]. Prolonged TLR signaling by Mtb has been shown to inhibit certain macrophage immune responses to IFN-γ, especially those related to MHC-II antigen presentation, which was believed to be a survival mechanism of Mtb in evading T cell responses [72]. Our findings indicate that this downregulation of MHC-II expression by MAP occurs irrespective of prior sensitization to IFN-γ and this feature was not observed in response to non-pathogenic *M*. *smegmatis*, indicating the potential for pathogenic mycobacteria to overcome the potentiating capability of IFNγ. This is likely to be associated with enhanced signaling via TLR2 as it has been shown previously by our group that increased TLR2 expression was observed only in response to MAP and not MS [54].

Our results agree with those of several previous publications on pathogenic mycobacteria [42, 73–80]. Although IFN-γ failed to overcome the inhibitory effect by pathogenic mycobacteria and its antigens on the parameters mentioned earlier, IFN-γ increased the secretion of pro-inflammatory cytokines IL-1α and TNF-α in response to MAP and 316v Ag respectively. It also enhanced the expression of the anti-inflammatory cytokine IL-10 significantly in response to MAP but decreased it in response to antigens. This leads us to believe that MAP can initiate a mixed M1/M2 polarization in macrophages, supporting our recently published data [81]. Also, antigens behave differently to whole bacteria in the way they interact with and induce innate immune responses and attempting to interpret the pathogenesis of a disease based on antigenic responses could be confounding. IFN-γ mediated increases in IL-1α and TNF-α may alter the cellular energy metabolic status due to their hypoglycaemic properties [82] resulting in the phenomenon of cellular polarisation characterised by decreased expression of antigen presenting molecules and co-stimulatory molecules such as MHC-II, CD80 and CD86 [83, 84]. Furthermore, although MHC-II expression was decreased even after IFN-γ pre-treatment, the impact of other cytokines or cellular effectors and the pathways involved would need to be verified. This has important implications as the notion that IFN-γ elicits solely a protective immune response in mycobacterial infections is a paradigm that developed with time, it is not an axiom and future studies would need to examine the relative contribution of this cytokine to inhibiting or possibly promoting mycobacterial persistence.

Based on our findings, we propose that although pre-stimulation with IFN-γ prior to encounter with viable mycobacteria may be advantageous in the initiation of selective protective immune responses it does not fully overcome the host immune inhibition initiated by pathogenic mycobacteria. This model system may be useful to investigate mycobacterial components that elicit optimal protective responses in relation to vaccine design. Under *in vivo* conditions, cells are likely to be exposed to a plethora of stimuli with synergistic interaction between different signaling mechanisms occurring simultaneously. Therefore, it would be interesting for future studies to assess the role of IFN-γ signaling *in vivo* in mycobacterial infection models or utilize 3D cell culture technology for *in vitro* approaches. This would help to gather critical information on IFN-γ signaling in macrophage function during infection with pathogenic mycobacteria and in response to vaccines.

## Supporting information

S1 Fig Titration of mycobacterial antigens. RAW 264.7 macrophages were pre-treated with IFN-γ (100 ng/mL) followed by incubation with 0–128 μg/mL of PPDB (A), 316v antigen (B), or PPDA (C). Nitric oxide was determined in supernatants harvested at 48 hours post-incubation. Data are the mean plus standard error of the means of six replicates from two individual experiments.
(TIF)

S2 Fig Effect of IFN-γ pre-sensitization on macrophage NFκB activation and translocation in response to mycobacteria. RAW 264.7 cells were incubated for one hour with media, live or heat killed MAP or *M*. *smegmatis* with or without pre-treatment with IFN-γ (100 ng/mL) for 30 minutes (E). The cells were stained for NFκB by immunofluorescence and images were captured using a fluorescence microscope. Image analysis and quantification was performed using using Image ProPlus^®^ software (F).
(TIF)

S3 Fig Gene expression in response to mycobacteria and antigens. RAW 264.7 cells were incubated with MAP, non-pathogenic mycobacterial strains, live (L) or heat-killed (HK) at an MOI of 100 to 1 or antigens, LPS (25 ng/mL), 316v or PPDB (100 μg/mL) for a period of 24 hours with or without pre-treatment with IFN-γ. Cells were harvested, RNA extracted and expression of genes assessed by the Quantigene 2.0 plex assay. Results are expressed as fold change compared to media control without pre-stimulation as a heat map (A) and as a table (B) for ease of comparison. Negative values indicate downregulation. Changes <1.5 fold were considered to be not regulated and are not colored.
(TIF)

S4 Fig MHC-II expression in response to mycobacterial antigens. RAW 264.7 macrophages were incubated with 316v Ag (100 μg/mL), PPDB (100 μg/mL) or Tuberculin (10 μg/mL) following pre-treatment with media alone, or IFN-γ (100 ng/mL) for one hour. Surface expression of MHC-II was detected at 24 hours post-incubation by flow cytometry. The median fluorescence value of the antibody minus the corresponding value for the isotype control was used to represent MHC-II expression. Data shown are the mean plus standard error of the mean for receptor expression for six replicate cultures from two separate experiments.
(TIF)

S1 File Supporting data (Panomics data). This file contains results for the gene expression changes described in Table 2.
(XLSX)
