# Supplementary figures and images for: IFN-γ fails to overcome inhibition of selected macrophage activation events in response to pathogenic mycobacteria
Source: PLoS One. 2017 May 15;12(5):e0176400. doi: 10.1371/journal.pone.0176400 (PMC5432162; doi:10.1371/journal.pone.0176400)

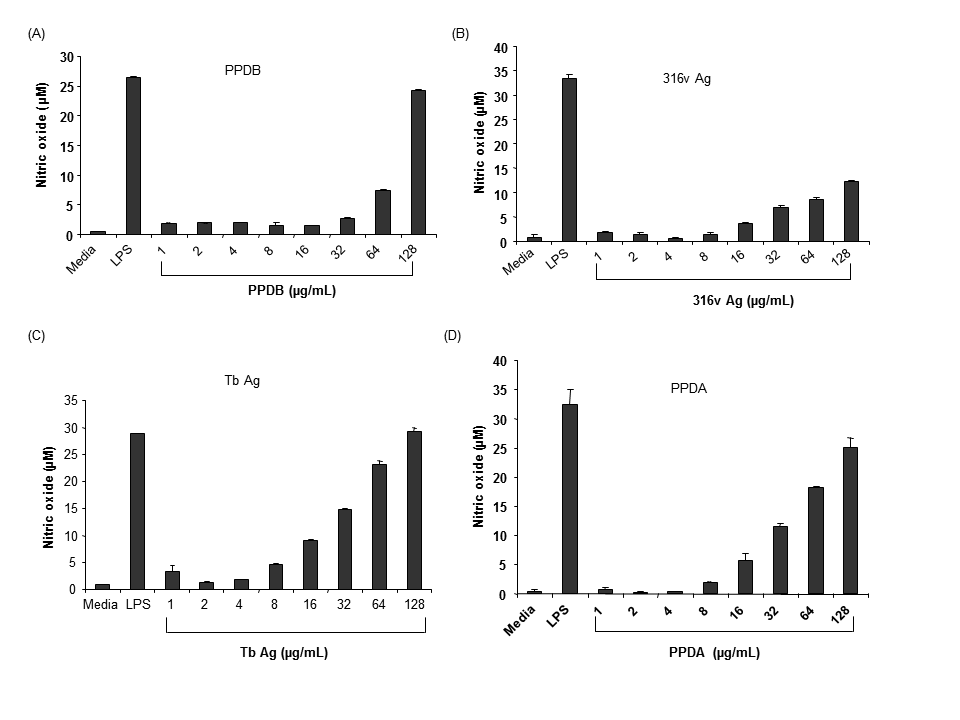

Supplement: S1 Fig — RAW 264.7 macrophages were pre-treated with IFN-γ (100 ng/mL) followed by incubation with 0–128 μg/mL of PPDB (A), 316v antigen (B), or PPDA (C). Nitric oxide was determined in supernatants harvested at 48 hours post-incubation. Data are the mean plus standard error of the means of six replicates from two individual experiments. (TIF) [file pone.0176400.s001.tif]

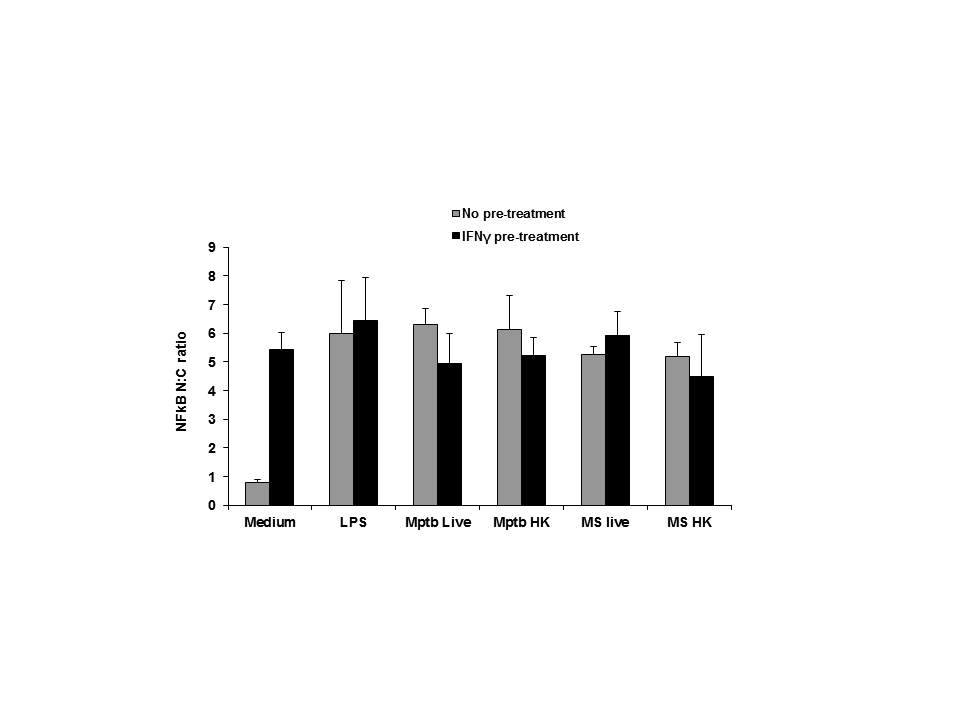

Supplement: S2 Fig — RAW 264.7 cells were incubated for one hour with media, live or heat killed MAP or M. smegmatis with or without pre-treatment with IFN-γ (100 ng/mL) for 30 minutes (E). The cells were stained for NFκB by immunofluorescence and images were captured using a fluorescence microscope. Image analysis and quantification was performed using using Image ProPlus® software (F). (TIF) [file pone.0176400.s002.tif]

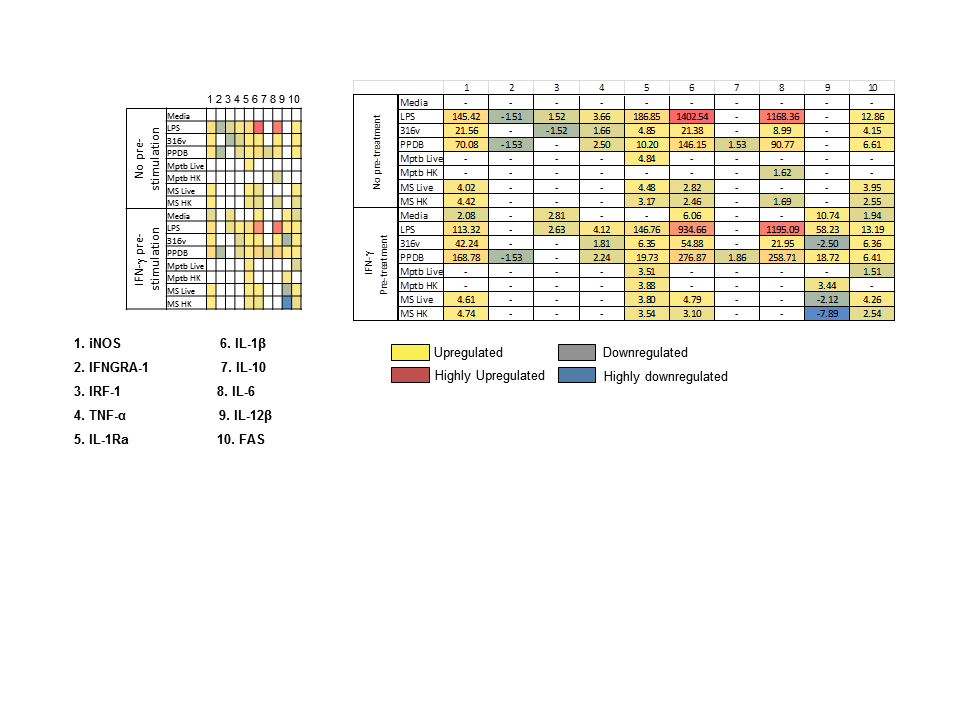

Supplement: S3 Fig — RAW 264.7 cells were incubated with MAP, non-pathogenic mycobacterial strains, live (L) or heat-killed (HK) at an MOI of 100 to 1 or antigens, LPS (25 ng/mL), 316v or PPDB (100 μg/mL) for a period of 24 hours with or without pre-treatment with IFN-γ. Cells were harvested, RNA extracted and expression of genes assessed by the Quantigene 2.0 plex assay. Results are expressed as fold change compared to media control without pre-stimulation as a heat map (A) and as a table (B) for ease of comparison. Negative values indicate downregulation. Changes <1.5 fold were considered to be not regulated and are not colored. (TIF) [file pone.0176400.s003.tif]

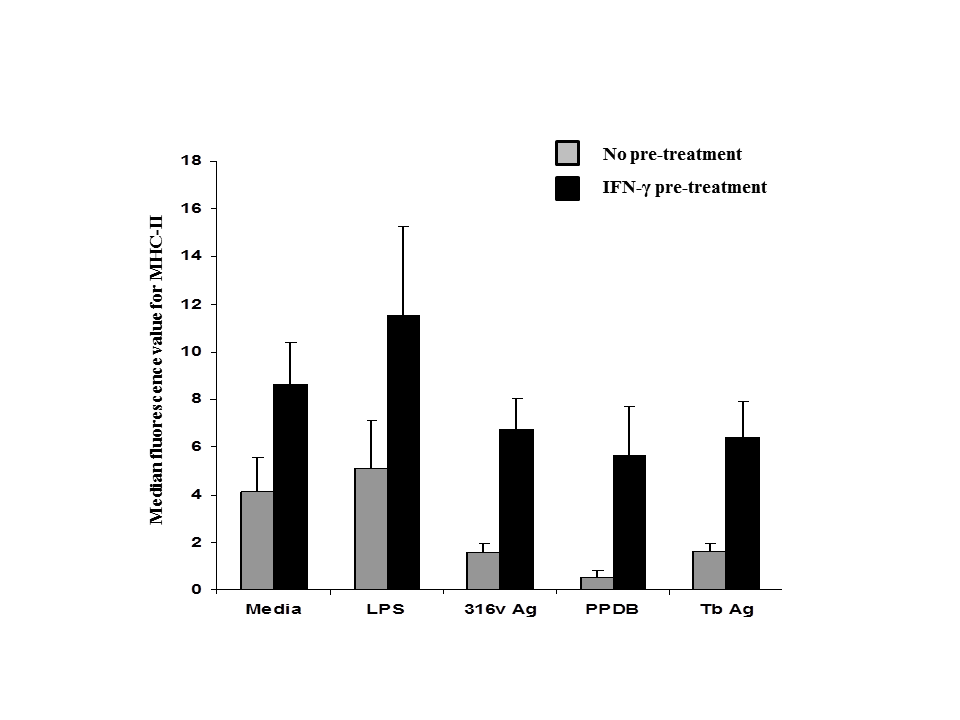

Supplement: S4 Fig — RAW 264.7 macrophages were incubated with 316v Ag (100 μg/mL), PPDB (100 μg/mL) or Tuberculin (10 μg/mL) following pre-treatment with media alone, or IFN-γ (100 ng/mL) for one hour. Surface expression of MHC-II was detected at 24 hours post-incubation by flow cytometry. The median fluorescence value of the antibody minus the corresponding value for the isotype control was used to represent MHC-II expression. Data shown are the mean plus standard error of the mean for receptor expression for six replicate cultures from two separate experiments. (TIF) [file pone.0176400.s004.tif]
